# Adaptation and validation of UNICEF/Washington group child functioning module at the Iganga-Mayuge health and demographic surveillance site in Uganda

**DOI:** 10.1186/s12889-020-09455-1

**Published:** 2020-09-01

**Authors:** Nukhba Zia, Mitchell Loeb, Dan Kajungu, Edward Galiwango, Marie Diener-West, Stephan Wegener, George Pariyo, Adnan A. Hyder, Abdulgafoor M. Bachani

**Affiliations:** 1 Johns Hopkins International Injury Research Unit, Health Systems Program, Department of International Health, Johns Hopkins University Bloomberg School of Public Health, 615 N. Wolfe Street, Suite E-8132, Baltimore, MD 21205 USA; 2 Washington Group on Disability Statistics, Hyattsville, MD USA; 3 Iganga-Mayuge Health and Demographic Surveillance Site, Makerere University School of Public Health, Kampala, Uganda; 4 Department of Biostatistics, Johns Hopkins Bloomberg School of Public Health, Baltimore, MD USA; 5 Department of Physical Medicine and Rehabilitation, Division of Rehabilitation Psychology and Neuropsychology, Johns Hopkins School of Medicine, Baltimore, USA; 6 Department of International Health, Johns Hopkins Bloomberg School of Public Health, Baltimore, MD USA; 7 Milken Institute School of Public Health, George Washington University, Washington, DC, USA

**Keywords:** Child disability, Adaptation, Validation, UNICEF/Washington group child functioning module, Iganga-Mayuge health and demographic surveillance site, Uganda, Africa

## Abstract

**Background:**

The UNICEF/Washington Group Child Functioning Module (CFM) assesses child functioning among children between 5 and 17 years of age. This study adapted and validated the CFM at the Iganga-Mayuge Health and Demographic Surveillance Site (IM-HDSS) in Uganda.

**Methods:**

This cross-sectional study was conducted between September 2018–January 2019 at the IM-HDSS. Respondents were caregivers of children between 5 and 17 years of age who were administered modified Washington Group short set (mWG-SS) and CFM. The responses were recorded on a 4-point Likert scale. Descriptive analysis was conducted on child and caregiver demographic characteristics. Exploratory factor analysis (EFA) assessed underlying factor structure, dimensionality and factor loadings. Cronbach’s alpha was reported as an assessment of internal consistency. Face validity was assessed during the translation process, and concurrent validity of CFM was assessed through comparison with disability short form.

**Results:**

Out of 1842 caregivers approached, 1439 (78.1%) participated in the study. Mean age of children was 11.06 ± 3.59 years, 51.4% were males, and 86.1% had a primary caregiver. Based on EFA, vision, hearing, walking, self-care, communication, learning, remembering, concentrating, accepting change, behavior control, and making friends loaded on factor 1 - “Motor and Cognition,” while anxiety and depression loaded on factor 2 - “Mood”. Cronbach’s alpha for the overall CFM was 0.899 (good internal consistency). Cronbach’s alpha for each extracted factor was excellent, motor and cognition (0.904), and mood (0.902). CFM had acceptable face validity. Spearman’s rank correlation between scores of CFM and modified WG short set was 0.51 (*p*-value < 0.001). The overall mean CFM score was 2.47 ± 3.82 out of 39. The mean score for Mood (1.35 ± 1.42 out of 6) was higher compared to Motor and Cognition (1.12 ± 3.06 out of 33). Comparing modified WG short set and CFM Likert responses, the percent agreement was greatest for “cannot do at all.”

**Conclusion:**

CFM is a two-factor, valid and reliable scale for assessing disability in Uganda and can be applied to other similar settings to contribute towards disability data from the region. It is an easy-to-administer tool that can help in deeper understanding of context-specific burden and extent of disability in children between 5 and 17 years of age.

## Background

Measurement of disability at the population level has been particularly problematic due to complexity of the disability phenomenon [1–3]. This has been further exacerbated, especially in low-and-middle-income countries (LMICs), by prevailing socio-cultural norms [4, 5]. In addition, the field of disability has evolved in its conceptualization of disability over the past several decades - moving from defining disability as a purely medical phenomenon to an interaction between impairments at the body level, in the context of a health condition as well as contextual factors specific to the environment in which the individuals live, making disability a complex phenomenon to assess [1, 2, 5, 6]. This has had a direct implication on population-based assessments of disability and its impact on individuals, families and the society. Furthermore, tools developed in high-income countries (HICs) may not be directly applicable to LMICs due to differences in context within which disability is assessed [3, 7, 8].

One of the main problems in understanding disability among children has been the lack of standardized, easy-to-use instruments that could be used to measure child disability [4, 9]. This has led to a lack of comparable disability estimates and hampers the development and evaluation of appropriate policies and programs to address the needs of children with disabilities [9]. Understanding not only the existence and type of disability but also its impact on children is therefore crucial as they go through different stages of growth and development, as disability influences their participation and functioning in the environment and society [3, 10].

Globally, one in 20 children under 15 years of age live with moderate or severe disability; about 90% of these children live in LMICs [3, 4, 11–13]. The overall estimated prevalence of moderate to severe disability in Africa is 15.3% [4]. It is important to point out that the 2011 World Report on Disability acknowledged that disability numbers are an underestimation and that reliable data on disability – prevalence, type, and causes- are lacking for most LMICs [4]. Over the past decade efforts have been made to generate meaningful disability data across various context and countries, accounting for the change in thinking related to disability and its definition as mentioned above. This has resulted in better understanding of the scope of disability across the globe, however, these efforts need to be further harmonized to ensure availability of valid and comparable disability data across the life span of an individual and especially among children.

Efforts to assess motor, cognitive, language, and social functioning in children has focused on using various tools often already used by clinicians. Most tools, for example the Mental Retardation Adaptive Behavior Scale or Griffith’s Scale of Mental Development, assess intellectual disabilities. The Ten Questionnaire (TQ) is a screening tool which assesses disability in children between 2 and 9 years of age and was found to be suitable for severe disabilities but tends to miss mild to moderate disabilities in populations. In addition, TQ was developed for use in a two-step process, where children are screened in step one followed by clinical assessment in step 2. However, step 2 is rarely done, especially in LMICs settings, due to limited resources available for clinical assessments [14, 15]. There is lack of data at the population level due to costs associated with acquiring these tools and their training requirements [7, 8, 16]. Moreover, countries that do have data on child disabilities lack a consistent and systematic approach for assessing such disabilities in children [3, 17]. Therefore, standardized methods to assess disability as part of national surveys has positive implications for a country; it allows monitoring of progress by making comparisons at national and international levels.

The Multiple Indicator Cluster Survey (MICS) administered by United Nations Children’s Fund (UNICEF) introduced a disability module for children in 2000 and collected data in 50 surveys from various LMICs. In 2011 UNICEF collaborated with the Washington Group on Disability Statistics (WG) to revise and develop a disability tool for assessing child functioning [18]. The WG, in collaboration with UNICEF, has undertaken the task to develop a set of questions that can be used to assess disability in children. These questions were developed based on the International classification of functioning, disability and health: children and youth version (ICF-CY) framework and comprise two sets of questions one for children between 2 and 4 years and the second for those between 5 and 17 years [19, 20]. The tool covers the domains of vision, hearing, walking, communication, learning, and behavior common to both age cohorts; as well as dexterity and playing for those 2–4 years of age and emotions, remembering behavior, concentration, coping with change and relationships for children 5–17 years of age. The questions were developed on the basis of the previous work that the WG had done to assess disability data in adults [21]. Children under 2 years of age were not included due to the challenges associated with assessing developmental delays in such young children. In addition, cultural norms tend to vary and can influence a child’s developmental milestones during infancy [18, 19]. The UNICEF/WG tool is an important data source for monitoring Sustainable Development Goals (SDGs). The tool can also be used to disaggregate data to help determine whether children with disability are disadvantaged compared to those without disability according to important outcomes like access to education; and to direct policy relevant activities that address child disability and may facilitate the monitoring of interventions [22]. As of now, the tool for children in the 5–17 years age group has not been validated in Africa (or Uganda).

This study aimed to adapt and validate the CFM applicable to children between the ages of 5–17 years at the Iganga-Mayuge Health and Demographic Surveillance Site (IM-HDSS) in Uganda. More specifically, this study developed a Lusoga version of CFM from its English version and conducted exploratory factor analysis (EFA) of the tool to assess its underlying factor structure, dimensionality, reliability and validity.

## Methods

### Study site

IM-HDSS is located in Eastern Uganda and covers the districts of Iganga and Mayuge. The site is part of the International Network for the Demographic Evaluation of Population and Their Health Network (INDEPTH) and was established in 2005 as a field research site for Makerere University [23–25]. About 38% of the IM-DSS is peri-urban and is located mostly around Iganga town; females comprise about half of the population. IM-HDSS has a crude birth rate of 21.1 live births per 1000 population and a crude death rate of 4.2 per 1000 population [24].

IM-HDSS follows over 94,000 individuals living in about 18,000 households. It conducts census level data collection two times per year on births, deaths, pregnancies and their outcomes, and in- and out-migrations [25]. In addition, IM-HDSS also periodically collects data on access to health services, causes of death, relevant socioeconomic and education data, non-communicable diseases and injuries [26]. Since 2005, 21 rounds of data collection have been completed as of June 2019 [23].

This study was nested within an ongoing parent study to pilot electronic data collection for injuries and disability in IM-HDSS. The main aim of the parent study was to strengthen local capacity to employ cutting-edge information and communication technology (ICT) for research and training on trauma, injuries, and disability. The purpose of the parent study was to pilot electronic versions of injury and disability data modules; these modules were implemented in paper format during previous studies conducted at IM-HDSS between 2008 and 2009 and subsequently were integrated into IM-HDSS; data was collected in three rounds [26, 27]. The IM-HDSS relies predominantly on paper-based data collection, [24, 25] and the process from data collection to entry into a database and analysis involves multiple steps [28]. However, the site is now transitioning to electronic data collection for efficient and timely availability of data for analysis. A pilot using tablet-based data collection was conducted in round 19 (April–June 2017), and was used as a sampling frame (see section below) for the current study on child disability.

### Study tools

Two tools were implemented as part of this study. Their details are provided below:

#### Modified Washington group short set (mWG-SS)

This study used a modified version of the Washington Group short set. The modified version includes the same domains as the original version except that remembering or concentrating was removed from the modified version and upper body mobility was added [21, 26]. mWG-SS has 6-questions for brief disability assessment that use a 4-level Likert scale (0 = no difficulty, 1 = some difficulty, 2 = a lot of difficulty and 3 = cannot do at all). Scores range from 0 to 18 such that the higher the score, the greater the difficulty. It focuses on activity limitations to identify individuals with disability and covers six domains: vision, hearing, walking, upper body mobility, self-care and communication. Previous studies conducted at the IM-HDSS and elsewhere found that it takes approximately 10 min to administer, and the questions are well understood by respondents [21, 26, 29, 30]. The main purpose of this tool is to identify individuals who, because of difficulties in the six basic activities, are at potential risk of limitation in their social participation (access to education, employment etc.,) if their environment is not accommodating. The WG-SS has been extensively tested both cognitively (15 countries) and in the field (five countries) [31, 32].

It is important to note that mWG-SS was already translated into Lusoga (the local language) and was implemented at IM-HDSS for disability assessment in individuals 5 years and above at the household level [26, 27]. The mWG-SS was first introduced at the IM-HDSS site in 2009, and since then it has been implemented three more times (2011, 2014 and 2017). Currently, adults (18 years and older) identified to have a disability based on mWG-SS are followed-up using a more detailed disability assessment tool to further characterize the implications of their activity limitation on different life domains. The WHO Disability Assessment Schedule 2.0 (WHODAS 2.0) is used for this purpose and was first implemented at the IM-HDSS in 2011, with another round conducted in 2017 [26, 27].

The data collection in round 19 (April – July 2017) was the first time IM-HDSS piloted electronic data collection of mWG-SS and WHODAS 2.0. Since the WHODAS 2.0 is only applied to individuals over 18 years, disability among children has not been further studied at the IM-HDSS. Thus, this study focused on this age group and is therefore an extension of the current disability work being done at IM-HDSS to allow for a better understanding of disability among children.

#### UNICEF/Washington group child functioning module (CFM)

This study utilizes a detailed CFM tool developed by the UNICEF/Washington Group on Disability Statistics [19]. CFM focuses on basic, everyday activities and has an expanded set of questions, beyond the mWG-SS, to assess functioning of a child. Like mWG-SS, it can be administered at the national level and allows for comparisons across time and countries. As recommended by the UNICEF/Washington Group, CFM was administered to caregivers of children previously identified to have disability using mWG-SS [14, 19, 33]. The tool was developed to cover the ICF-CY domains of vision, hearing, mobility, self-care (including feeding and dressing), communication, learning, remembering, concentration, accepting change, behavior, making friends, feeling anxiety, and feeling depression [20]. It comprises 24-questions over 13 domains of functioning with responses on a 4-level Likert scale (0 = no difficulty, 1 = some difficulty, 2 = a lot of difficulty and 3 = cannot do at all). These questions result in 13 domains with scores ranging from 0 to 39; the higher the score, the greater the disability. It takes about 20–25 min to complete.

### CFM translation from English to Lusoga

**Fig. 1:**
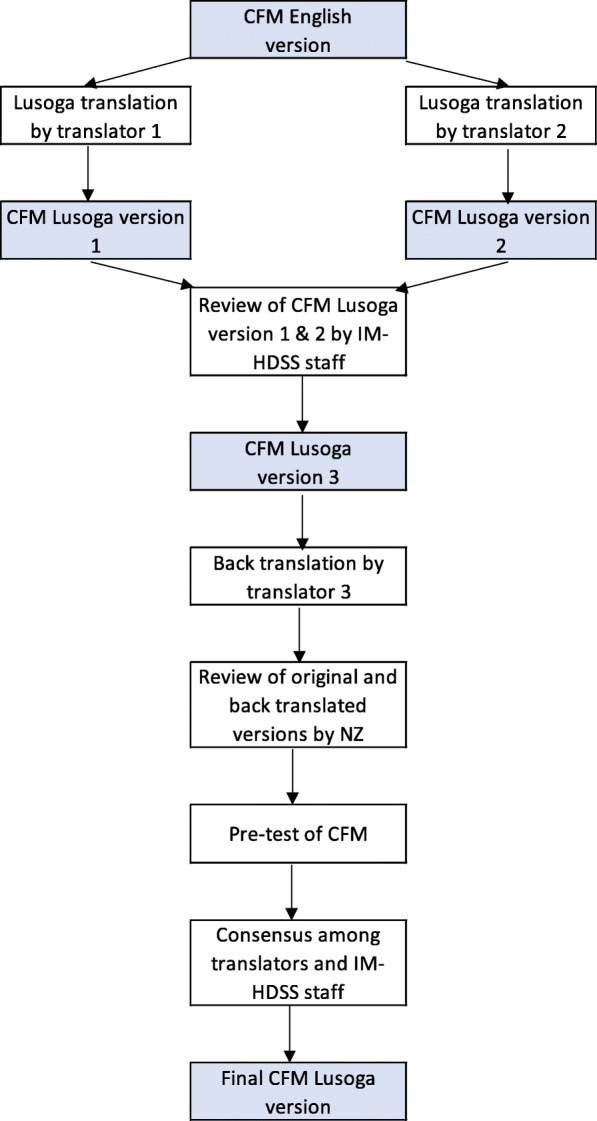
Flow chart of translation process of UNICEF/WG Child Functioning Module (CFM)

The CFM has been through several rounds of cognitive and field testing already; however, it has not been validated in Uganda [20]. CFM was translated to Lusoga by two independent translators well-versed in English and Lusoga and aware of the local context. The Lusoga translations were reviewed by one of the IM-HDSS field coordinators to check for any discrepancies between the translated versions. The Lusoga version was then back translated by a third translator. Author (NZ) compared the English translation with the original tool to identify any inconsistencies. The tool was pre-tested with 46 respondents to ensure that questions and responses were clearly stated and comprehensible for respondents. The final version used for data collection was developed in consultation with IM-HDSS field coordinators and supervisor (Fig. 1). The translation and back-translation helped in assessing face validity of the Lusoga version of CFM.

### Study design and respondents

This was a *cross-sectional study* conducted between September 2018 – January 2019. Respondents were caregivers of children between 5 to 17 years of age who were administered mWG-SS and CFM. At the time of the study, there were 35,062 children between the ages of 5–17 years who were residing in the IM-HDSS.

### Sampling frame

Sampling frame for this study was drawn from household and individual listings available from the latest IM-HDSS database specifically for rounds 19 and 20. Data from a pilot conducted as part of round 19 served as basis for identifying children with disabilities who were between 5 and 17 years of age. This was done using data from mWG-SS that was administered at the household level. A total of 377 children between the ages of 5–17 years were identified to have some form of disability based on round 19 mWG-SS data. Their IDs were then confirmed for active status in round 20, which had been completed 4 months (May 2018) before the beginning of this study (September 2018). Based on the round 20 check, 342 children out of 377 from round 19 were found to have active IDs (29 children were more than 17 years, one had died, 4 had moved to another location within IM-HDSS, and one had moved out of IM-HDSS). Active IDs mean that these children were present at the IM-HDSS site as of round 20 (the latest round before data was collected for this study); hence, all these 342 children were included in this study.

In addition to children with disability, for validation of CFM, a sample size of 1273 was computed to detect a difference of 1% between the groups of children with versus without disability, assuming alpha of 5% and power of 80%. This resulted in a total sample size of 1615: 1273 children without disability and 342 with disability. However, the sample size of children without disability was increased to 1500 to account for non-availability, refusals, and out-migrations from the site. At the time of this study, 35,062 children (excluding 342 with disability) between 5 and 17 years of age were residing at the IM-HDSS. Stratified (sex) sampling proportionate to the population size of children without disability (*n* = 35,062) was performed after removing the 342 children with disability from the round 20 active IDs. The formula used for sample size calculation was:

$$ \frac{Total\ sample\ size\ required}{Population\ size}\times Stratum\ size $$

Based on the above formula, a stratified sample of 737 males and 763 females for a total of 1500 children without disability was calculated.

**Fig. 2:**
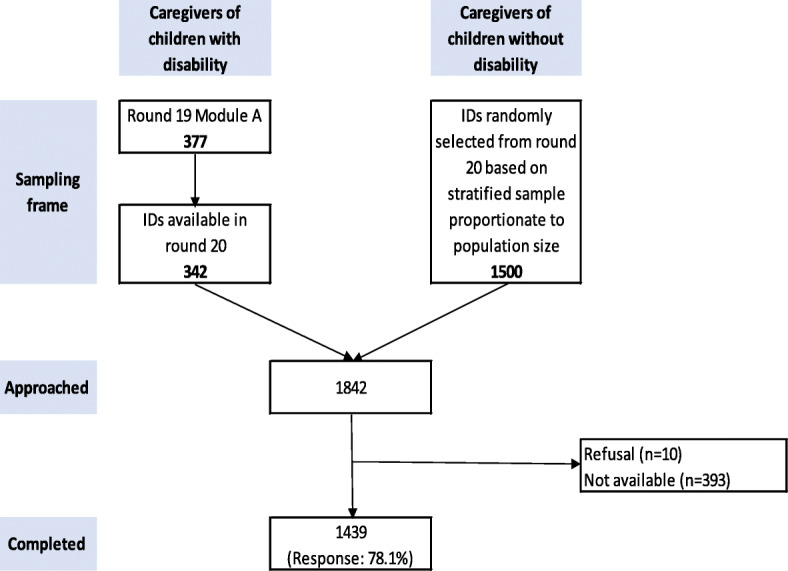
Flow chart of caregiver enrollment for Child Disability study

A random list of IDs was drawn from each stratum using STATA version 14 [34]. Thus, sample for this study included 342 children with disabilities and 1500 children without disabilities, giving a total of 1842 children whose caregivers were approached to participate in the study. A unique study ID was assigned to all 1842 children included in the sample. Only one child per household was selected. Figure 2 gives enrollment of caregivers.

It is important to note that the distinction between children with disability and without disability was made for sampling purpose to ensure that sample for this study does not miss children with disabilities. The analysis for this study was conducted on the pooled sample of individuals who agreed to participate in the study.

### Sample size

The sample size calculation for exploratory factor analysis (EFA) was based on two rules [35, 36]. The first rule suggests taking a ratio of subjects to items: a ratio of 5–10 respondents per item to about 300 respondents [37–39]. CFM has a total of 24 questions. Going by the 5:1 or 10:1 rule, a sample size in the range of 120–240 was obtained. The second rule suggests having a minimum total sample size; according to this, a sample size of 100 is poor, 200 is fair, 300 is good, 500 is very good, and 1000 or more is excellent [40]. The suggested minimum size is between 400 and 500 participants. Thus, based on these rules, this study had a sufficient sample size (*n* = 1842) to conduct EFA. Having a larger sample size increases stability of factor analysis results and conclusions. It also helps in replication of results to assess generalizability of the tool [35].

### Data collection and management

After obtaining oral informed consent, data were collected through face-to-face interviews with caregiver using a tablet-based platform that was developed and pre-tested using available local resources at the IM-HDSS. The platform included English and Lusoga versions of questions, which were developed in Microsoft Excel .xls format and uploaded to KoBoToolbox (https://www.kobotoolbox.org/) for data collection. Questions had a check box and free text entry formats to enter responses. Questions were designed to allow skip patterns where appropriate, and mandatory fields were also marked. This ensured that there were no missing data for mWG-SS and CFM. In order to reduce workload related to entry of IM-HDSS IDs, the unique study ID was linked with the IM-HDSS IDs at the backend in order to address issues related to errors in ID entry. The Kobo app was downloaded to android tablets to allow for data collection using a user-specific password. These forms were accessible in the field during data collection and did not require internet or Wi-Fi connection. Once an interview was completed, the form was saved on the tablet. Field supervisors checked saved forms at the end of the day, and completed forms were uploaded daily using office Wi-Fi connection to a cloud server. The electronic forms were submitted to a secure, encrypted cloud server with no copy available on the tablet after submission to the cloud server. The server was only accessible to authorized study team members at IM-HDSS and Johns Hopkins Bloomberg School of Public Health (JHSPH). This ensured data confidentiality and security. Data were downloaded daily from the server in MS Excel (.xls and .cvs format).

### Data analysis

#### Descriptive analysis

Descriptive analysis was conducted to assess demographic characteristics of children and their caregivers. Binary and categorical variables are reported in percentages and mean with standard deviation as well as median and interquartile range (IQR) are reported for continuous variables.

#### Exploratory factor analysis (EFA)

Disability is a latent variable and cannot be measured directly. Through CFM, the 13 domains mentioned above indirectly measure disability. Exploratory factor analysis (EFA) is a data reduction method and is also used for construct validity [35, 41]. It identifies an underlying factor structure of a set of directly measured variables and an associated number of latent constructs or factors. EFA also helps to determine if there is only one or more underlying latent constructs being assessed by the domains.

The eligibility to use items for EFA is determined by using two tests. First, the Bartlett’s test of sphericity is used to test the null hypothesis that the items are not correlated. To perform EFA, the Bartlett’s test needs to be statistically significant, which means that there are sufficient intercorrelations between variables to conduct the factor analysis [42]. Second, the Kaiser–Meyer–Olkin (KMO) provides a measure of sampling adequacy. KMO identifies items that are related and also provides unique information on the factors identified by the EFA. Higher values of KMO indicate overlap of variance between variables but not to the point of hindering the analysis due to multicollinearity [43, 44]. As guidelines, KMO of 0.00–0.49 is unacceptable, 0.50–0.59 is miserable, 0.60–0.69 is mediocre, 0.70–0.79 is middling, 0.80–0.89 is meritorious, and 0.90–1.00 is marvelous. Having larger values is better and indicates a measure of overall or shared variance between pairs of variables [43]. Since Likert scale response for CFM is considered in this analysis, polychoric correlation of the domains was assessed. Polychoric correlation gives the measure of association between ordinal variables. Its value range is between 1 and − 1. A value of 1 or − 1 mean perfect correlation while value of 0 means no correlation [45].

EFA has three main steps: (a) determining the number of factors, (b) selecting an extraction method, and (c) choosing a rotation method. Criteria for retaining factors included: (a) Kaiser criterion of an eigenvalue of greater than 1, and (b) number of factors to the left of scree plot elbow. Eigenvalue shows total variance accounted for by each factor. It accounts for most of the variance explained by the underlying factor [35, 44].

There are various extraction methods in EFA that give factor loadings for every item on every extracted factor. The method used for this study was iterative factor analysis, which does not require any distributional assumption for the underlying factors. In this study, EFA was performed using promax rotation (oblique rotation), which assumes that the extracted factors are correlated. Factor loadings of ≥0.30 are considered for EFA. The number of factors retained, their respective eigenvalues, uniqueness (variance unique to the variable and not explained by other variables) and estimation method for model fit are reported for the EFA.

#### Reliability

The reliability of CFM was calculated using Cronbach’s alpha, which is a measure of internal consistency. Cronbach’s alpha of ≥0.9 is excellent, ≥0.8 is good, ≥0.7 is acceptable, ≥0.6 is questionable, ≥0.5 is poor and < 0.5 is unacceptable [39]. Cronbach’s alpha for the extracted factors is also reported [46].

#### Validity

Face validity was assessed through translation and back-translation process as well as pre-testing of CFM. Concurrent validity, a type of criterion validity, was assessed by administering CFM and mWG-SS concurrently. Criterion validity allows assessment of the relation between two tools or measures that measure the same construct. In this study, CFM was compared with disability short form, which was already validated at IM-HDSS and has been implemented several times at the IM-HDSS site [26, 27]. Since CFM and mWG-SS were administered at the same time, concurrent validity (type of criterion validity) is reported using the Spearman’s rank correlation coefficient. The hypothesis was that those with high mWG-SS scores will also have high CFM scores [35, 47]. Spearman’s rank correlation coefficient is reported when the response variable is ordered and there is a monotonic relation between the variables. Its value ranges between − 1 to + 1. The values are interpreted as “very weak” for values between 0.00–.19, “weak” for 0.20–0.39, “moderate” for 0.40–0.59, “strong” for 0.60–0.79, and “very strong” for 0.80–1.0 [48]. In addition to assessing correlation between CFM and mWG-SS scores, Likert scales responses of CFM and mWG-SS were also compared using percent agreement, which is the percentage having the same CFM response for a given mWG-SS response. Cohen’s Kappa was also calculated to account for chance agreement between the two tools. Its value varies from 0 to 1 where 0 mean reflects chance agreement and 1 mean reflects perfect agreement. Values between 0.1–0.20 show slight agreement, 0.21–0.40 is fair agreement, 0.41–0.60 is moderate agreement, 0.61–0.80 is substantial agreement, and 0.81–0.99 is near perfect agreement [49, 50]. Data analysis was conducted using STATA version 14 [34].

## Results

### Descriptive analysis

Out of 1842 caregivers approached, 1439 (78.1%) respondents were available for this the study (Fig. 2) and were included in this analysis. The mean reported age of children was 11.06 ± 3.59 years; 51.4% were males. Over half the children had not completed their vaccinations. Over half of the children belonged to a nuclear family system, and about a quarter were in the poorest wealth quintile (Table 1). More than 80% had a primary caregiver, with mother being the most common caregiver. Average age of the primary caregiver was 41.15 ± 11.69 years, with education up to primary (59%) (Table 2).

**Table 1:** Demographic characteristics of children (*n* = 1439)

Child characteristics n (%)	
Average age in years (mean ± SD)	11.06 ± 3.59
**Age groups**	
5–10 years	657 (45.66)
11–14 years	467 (32.45)
15–17 years	315 (21.89)
**Sex**	
Male	739 (51.36)
Female	700 (48.64)
**Immunization status**	
Complete	557 (38.71)
Not complete	790 (54.90)
No vaccination done	92 (6.39)
**Currently in school**	
Yes	1272 (88.39)
No	167 (11.61)
**Have a primary caregiver (yes)**	1239 (86.10)
**Family system**	
Single parent	138 (9.59)
Nuclear	743 (51.63)
Joint	555 (38.57)
**Household wealth quintile (*n*** **= 1389)**	
Poorest	389 (28.01)
Poorer	299 (21.53)
Poor	325 (23.40)
Less poor	248 (17.85)
Least poor	128 (9.22)

**Table 2:** Demographic characteristics of primary caregivers (*n* = 1239)

Caregiver characteristics n (%)	
**Caregiver relation with child**	
Mother	770 (62.15)
Father	164 (13.24)
Sibling	13 (1.05)
Uncle/Aunt	58 (4.68)
Grandparent(s)	173 (13.96)
Others	61 (4.92)
**Average age (mean ± SD)**	41.15 ± 11.69
**Sex**	
Male	219 (17.68)
Female	1020 (82.32)
**Education level**	
None	168 (13.56)
Primary	731 (59.00)
Lower secondary	250 (20.18)
Upper secondary	12 (0.97)
Other (university/vocational)	78 (6.30)
**Occupation**	
Farmer	679 (54.80)
Shopkeeper	281 (22.68)
Housewife	120 (9.69)
Professional	55 (4.44)
Boda boda driver	13 (1.05)
Unemployed	30 (2.42)
Others	61 (4.92)

### Exploratory factor analysis

The Bartlett’s test for sphericity was statistically significant (*p*-value < 0.001), showing that items are not correlated. KMO for sampling adequacy was 0.898, which is meritorious. Polychoric correlation showed that all domains are positively correlated, and most were statistically significant. Very strong correlation was found between self-care, communication, learning, remembering, concentration, accepting change, behavior control, and making friends, with correlations ranging between 0.50–0.95. The correlation between anxiety and depression was 0.89.

**Fig. 3:**
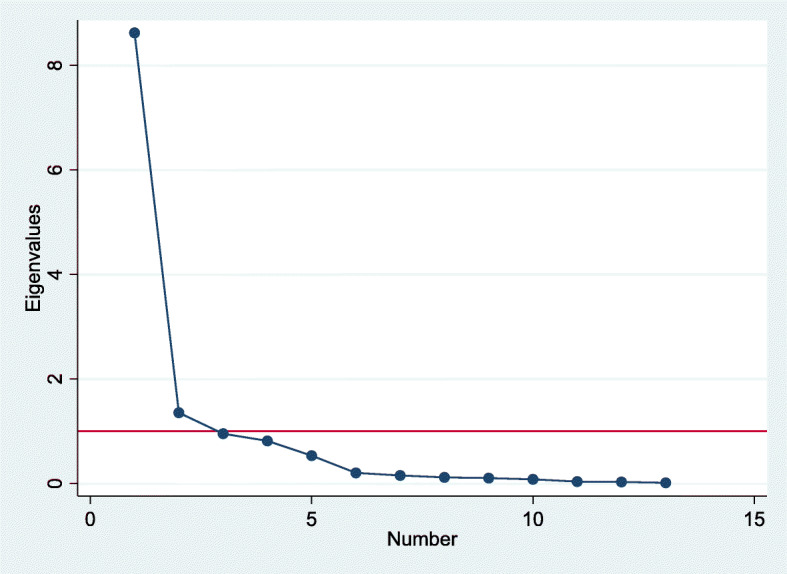
Scree plot based on exploratory factor analysis for selection of retained factors

Based on eigenvalue criteria of values > 1 and scree plot (Fig. 3), two factors were retained using principal components analysis. The first factor had an eigenvalue of 8.62, and the second had an eigenvalue of 1.35, explaining 66.32 and 10.41% of the total variance respectively. Together, the first two factors explain 76.73% of the total variance. Pattern matrix of rotated factor loadings showed that vision, hearing, walking, self-care, communication, learning, remembering, concentrating, accepting change, behavior control, and making friends loaded on factor 1 (Motor and Cognition), while anxiety and depression loaded on factor 2 (Mood) (Table 3).

**Table 3:** Factor loadings and uniqueness of UNICEF/WG Child Functioning Module (CFM) in Ugandan context

Domains	Factor 1: Motor and cognition	Factor 2: Mood	Uniqueness
Vision	0.30		0.91
Hearing	0.54		0.73
Walking	0.71		0.45
Self-care	0.79		0.21
Communication	0.88		0.14
Learning	1.00		0.10
Remembering	0.99		0.15
Concentration	0.95		0.05
Accepting change	0.93		0.12
Behavior	0.87		0.16
Making friends	0.86		0.08
Feeling anxiety		0.86	0.22
Feeling depression		1.00	−0.00

### Reliability

Cronbach’s alpha for the CFM was 0.899, showing good internal consistency. Cronbach’s alpha for each extracted factor was excellent – 0.904 for Motor and Cognition, and 0.902 for Mood. The Cronbach’s alpha for mWG-SS was 0.742, showing acceptable internal consistency.

### Face validity

No major modifications were made in CFM as a result of its translation and back-translation. During the pre-test, none of the participants reported any difficulty in understanding the questions and answer responses.

### Concurrent validity

**Fig. 4:**
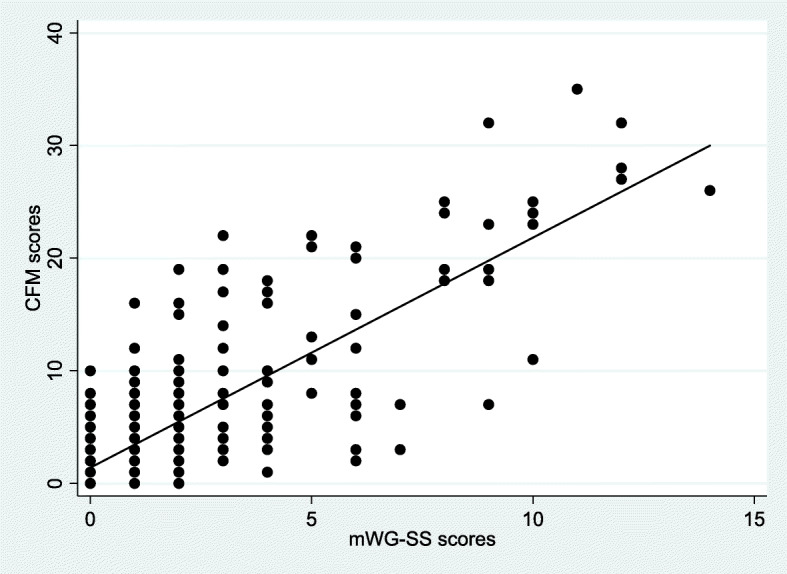
Scatterplot showing monotonic relationship between scores of UNICEF/WG Child Functioning Module (CFM) and modified Washington Group short set (mWG-SS)

Spearman’s rank correlation between CFM and mWG-SS scores was 0.51 (*p*-value < 0.001), showing a moderate positive correlation (Fig. 4). Table 4 compares Likert responses between CFM and mWG-SS and shows that the percent agreement was greater for “cannot do at all.” However, agreement for “some difficulty” and “a lot of difficulty” was around 70%. The observed overall percent agreement between CFM and mWG-SS was 48.02% and kappa was 0.219 (standard error: 0.014), showing minimal agreement between the two tools.

**Table 4:** Comparison between modified Washington Group short set (mWG-SS) and UNICEF/WG Child Functioning Module (CFM)

UNICEF/WG Child Functioning Module (CFM)	Modified Washington Group short set (mWG-SS)				
	None	Some	A lot	Cannot do at all	Total
**None**	458 (41.04)	4 (1.75)	0	0	462
**Some**	521 (46.68)	162 (70.74)	9 (12.68)	0	692
**A lot**	125 (11.20)	53 (23.14)	49 (69.01)	1 (4.35)	228
**Cannot do at all**	12 (1.08)	10 (4.37)	13 (18.31)	22 (95.65)	57
**Total**	1116	229	71	23	1439

## Discussion

To the best of our knowledge, this is the first study that adapted and assessed validity and reliability of CFM in Uganda. The analysis shows that the questions listed in the tool represent the underlying construct of disability through two factors – Motor and Cognition, and Mood— and 13 domains - vision, hearing, walking, self-care, communication, learning, remembering, concentrating, accepting change, controlling behavior, making friends, anxiety, and depression – all of which focus on functional disability, the underlying construct being assessed by the CFM tool. The UNICEF/Washington Group CFM for 5–17-year-old children was validated in school settings in Fiji in 2015 with the objective to determine if it can be used by teachers to identify children at risk of disability for timely referral for further assessment and interventions. That study conducted diagnostic accuracy testing to determine optimal cut-offs of scores to predict impairment in seeing, hearing and walking and found that “some difficulty” has optimal level of sensitivity and specificity compared to “a lot of difficulty” to assess disability status of children [51]. The analysis in the Fiji study is presented at domain level. It does not present results related to adaptation of the tool in Fijian context [51]. However, this study shows that CFM as a complete tool can be adapted into Ugandan context and other similar context and is valid to assess level of functioning in children. The Washington Group has suggested using cut-off approach (cut-offs are defined using Likert responses to categorize levels of disability for example a person with “cannot do at all” on one domain is categorized to have severe disability). However, their recent work is looking to use total raw scores based on Likert scale responses of CFM and mWG-SS (WG will make this work available to public in the Fall 2020). We therefore used total raw scores to replicate their work to validate the CFM in Uganda. Although this approach undermines the disability severity categorization, however follow-up to this work includes use of the cut-off approach [52].

CFM showed good overall internal consistency and excellent factor-level internal consistency. Its correlation with mWG-SS was moderate, while kappa agreement was minimal. This could be because CFM includes additional domains related to cognition (learning, remembering, concentrating, accepting change, controlling behavior, making friends, anxiety, and depression) which are not covered in mWG-SS – an illustration of the limitations of the mWG-SS among children. In addition, mWG-SS assesses upper body mobility, which is not assessed in CFM. However, CFM is well-suited for implementation within the Ugandan context. This has great implications for furthering the current discussion around lack of comparable disaggregated disability data at national, regional and global levels to monitor disability-related SDGs [12, 17, 30]. Disability has many manifestations, and each type of disability can be assessed separately. However, for effective interventions at the community level, it is important to understand the overall prevalence of disability and account for multiple disabilities in an individual. The focus of CFM is on assessing physical disability in children and is not applicable to children with intellectual disabilities due their unique needs.

Field testing of the initial version of UNICEF/WG tool for children 5–17 years of age was conducted in two rounds in several different settings, including India, Belize, Oman, Montenegro, and the USA, resulting in development of a final version that was used for this study [14, 31, 33, 53]. Like this study, the main respondents of the tool during field testing were caregivers of children [54]. A study from South Africa has used the CFM for 2–4 years old children in its 2011 General Household Survey and National Census to generate epidemiological evidence on child disability and to identify types and extent of disabilities [55].

Disability is considered to be on a continuum – from mild to severe. In order to use disability as an outcome variable, several different recommendations have been put forward which suggest using disability as a binary or categorical outcome [18, 51]. Categorization of disability into binary categories may result in loss of important information. It is important to understand the concept of heterogeneity associated with disability measurement. People with different degrees and types of disability may face different degrees and types of barriers. Combining all people into one group of “people with disabilities” can mask different impacts [2]. Changing the cut-off will change the measured impact. Most severe cut-offs will show the biggest difference in outcome between children with and without disabilities, while the opposite is true for less severe cut-offs, which will raise prevalence but show the smallest difference in outcome [33]. This has practical implications where interventions are planned, implemented and monitored for their impact and usefulness. This is even more crucial in LMICs, where limited resources hamper efforts to address needs of individuals with disabilities.

Some of the advantages of CFM are as follows. First, it does not require a clinician for administration and can be implemented easily by field staff or community workers [55]. Second, having a standardized and validated tool for settings like Uganda is very much needed for good quality, reliable, and accessible data to help with monitoring of SDGs. CFM has the benefit of use by multiple sectors – health, finance, education, and transport – to ensure inclusion of children with disability. Third, CFM was implemented as an electronic, tablet-based data collection system in a semi-urban/rural setting, which allowed for timely, efficient data capture and transfer. There were built-in validation mechanisms that allowed data quality checks. Such systems ensure timely availability of data, which can be shared with relevant stakeholders for planning purposes. Fourth, CFM is specific for children and allows for proxy responses from mothers/caregivers. The questions are structured such that they account for the growing needs of children and age-appropriate activities by asking questions like, “in comparison with children of the same age….” This also helps to consider local context [14, 31]. Fifth, the translation process and feedback from IM-HDSS and participants of the pre-test did not require many changes in the question structure and understanding of words and response options. This favors the use of a standard tool across different contexts to collect internationally comparable disability data.

Some limitations of the study are as follows: First, this was a cross-sectional study and doesn’t allow an assessment of changes in disability level over time. This is a crucial consideration in disability research and requires data systems that have the capacity to follow-up on children with disabilities to assess their growing needs along with their disability-specific needs. Second, merely collecting data on children with disability using mWG-SS and CFM is not enough. This study lacked resources to have clinical follow-up on children identified as having disability in the CFM domains. This needs to be addressed in future work at IM-HDSS in order to provide liaison mechanisms between IM-HDSS and the local district health office and healthcare facilities. Third, comparison between CFM and mWG-SS was performed using total raw scores to keep the analysis as close as possible to the responses received from the respondents for this study. Using total raw scores based on Likert responses assumes that the response categories are equidistant which is a limitation and will be addressed in our future work on disabilities in children in Uganda. Domain level comparison was only possible for seeing, hearing, walking, self-care and communication. Fourth, CFM lacks questions on upper-limb mobility as was also noted by Sprunt et al. [51] although the self-care question does cover some upper body activity. Perhaps future work can add this as one of the domains. This is an important domain to consider due to its implication on fine and gross upper-limb movements and opportunities for learning and education for these children. Fifth, there may be bias in how caregivers responded to questions if their child had disability [31]. This might have influenced their interpretation of the question, or they might have changed their responses to a more desirable option with the hope for monetary support or some gain in case the child is shown to have disability. In addition, for questions mentioning comparison to “other child of same age…,” the caregiver could have either compared to other children with disability or to other children without disability, which could result in biased responses based on disability status of the child [31]. Sixth, this paper focuses only on presenting validation and adaptation results. Data on type and extent of disability will be presented in a forthcoming under review paper on factors associated with disability [56]. Seventh, strict cut-off criteria (≥0.3) for factor loadings was used for this analysis. However, given lower factor loading for vision (0.3) and hearing (0.54), there is a possibility of a third factor. This needs to be explored in future research on CFM.

## Conclusion

CFM is a two-factor, valid and reliable scale for assessing disability in Uganda and other similar contexts. It is an easy-to-administer tool that helps in deeper understanding of context-specific burden and type of disability in children between 5 and 17 years of age. This standardized tool contributes to the global effort towards collection of meaningful and contextually relevant disability data that can be collected at national and sub-national levels in a timely manner to generate evidence for policy-makers, and for monitoring and evaluation of interventions.

## Data Availability

The dataset generated and analyzed during this study are not publicly available due IM-HDSS data policy but are available from the corresponding author on request.

## Abbreviations

CFM: UNICEF/Washington Group Child Functioning Module
EFA: Exploratory factor analysis
HICs: High-income countries
ICF-CY: International classification of functioning, disability and health: children and youth version
IM-HDSS: Iganga-Mayuge Health and Demographic Surveillance Site
INDEPTH: International Network for the Demographic Evaluation of Population and Their Health Network
KMO: Kaiser–Meyer–Olkin test for sampling adequacy
LMICs: Low-and-middle-income countries
MICS: Multiple Indicator Cluster Survey
mWG-SS: Modified Washington Group short set
SDGs: Sustainable Development Goals
TQ: Ten Questionnaire
WHODAS 2.0: WHO Disability Assessment Schedule 2.0
WG: Washington Group
